# History of medical body: Demystifying the continuum of psychiatry and psychoanalysis

**DOI:** 10.1192/j.eurpsy.2021.1038

**Published:** 2021-08-13

**Authors:** V. Gairola

**Affiliations:** School Of Human Studies, Ambedkar University Delhi, Delhi, India

**Keywords:** psychiatry, Psychoanalysis, Medical Body

## Abstract

**Introduction:**

The aim of this paper lies in demystifying, historicizing, and de-alienating the relationship between psychiatry and psychoanalysis. Both psychiatry and psychoanalysis inform each other and are informed by each other in various ways which are on one hand similar and on the other hand unique. Medicine can be seen psychoanalytically, and a presenting complaint to a psychoanalytic psychotherapist can be seen in psychiatric terms.

**Objectives:**

This paper theorizes the ‘and’ between psychiatry and psychoanalysis. What is that bridging telling us? There is already an invisible ‘and’ which joins psychiatry and psychoanalysis even before this visible ‘and’ was placed in between them. The effort here is not to undermine the difference. It is to be aware that the thing which separates is also a thing which connects. In other words, each separation is a link. It is to understand how psychiatrists have contributed to the method and practice of psychoanalysis and visa versa. It is about witnessing the continuum that is ever-present between psychiatry, psychology, and psychoanalysis.

**Methods:**

This research used primary sources like books and articles to historicize the psychiatric conceptualization of the medical body.

**Results:**

It shows how psychiatry and psychoanalysis inform each other and are informed by each other. To locate the historical conceptions which are still ever-present in modern psychiatry. How the 18th-century division between normal and abnormal is based on an older division between good and evil.

**Conclusions:**

Good and evil continue to operate in the realm of psychiatry and the confessional becomes a prime source for psychiatry and psychoanalysis.

